# Two-step heat fusion kinetics and mechanical performance of thermoplastic interfaces

**DOI:** 10.1038/s41598-022-09573-3

**Published:** 2022-04-05

**Authors:** Shijun Wang, Jiaxin Shi, Takayuki Shimizu, Jun Xu, Zhiping Xu

**Affiliations:** 1 Applied Mechanics Laboratory, Department of Engineering Mechanics and Center for Nano and Micro Mechanics, Tsinghua University, Beijing, 100084 China; 2 Department of Chemical Engineering, Tsinghua University, Beijing, 100084 China; 3 Strength Research Department, Research & Innovation Center, Mitsubishi Heavy Industries Ltd., Nagoya, 455-8515 Japan; 4 National Center for Nanoscience and Technology, Beijing, 100190 China

**Keywords:** Composites, Coarse-grained models, Polymers

## Abstract

Thermoplastic polymers and composites are ubiquitous in the industry for their reshaping and fusing capabilities at elevated temperatures. The quality of heat-fused thermoplastic interfaces is of great concern for adhesion, coating, and welding applications, especially those between dissimilar materials. Kinetic evolution of the microstructures defines the mechanical performance of heat-fusion thermoplastic interfaces, which is studied here using polyethylene and polypropylene as an example. Key factors such as the viscosity and compatibility of polymers and the time and temperature of fusion are discussed by combining molecular-level simulations and structural-level hot-compression experiments. Inter-diffusion and entanglement of polymer chains are identified as the two elementary kinetic steps of the fusion, which dominate the control on the stiffness and strength of the interfaces, respectively. Experimental data shows that the quality of fused interfaces can be improved by reducing the viscosity and the interaction parameter. Following the same set of time-scaling relations as identified in the simulations, the two-step characteristics and their effects on the stiffness and strength are experimentally validated. Both simulation and the experiment results show that Young’s modulus of fused interfaces recovers faster than the strength that is controlled by polymer entanglement to a large extent, rather than diffusion. These findings add insights into the design of fusion processes, laying the ground for the applications of thermoplastic polymers and composites.

## Introduction

Plastics and their composites are one of the most widely-applied synthetic materials in the world, with over one third of a billion tones of global production every year^1–4^. First appeared in early 20-th century and not commercially applied until 50s, plastic products are growing extraordinarily, especially in the packaging market^2^, electrical and electronic products^5^, buildings and constructions, textiles, transportation, and medical equipment^6^. The high demands of reusable, safe, economic and functional plastic products accelerate the evolution of plastic processing technologies. In contrast to the thermosetting polymers, thermoplastics are convenient for their reshaping and recycling capabilities, making them ideal candidates for emergent applications in adhesives^7^, coating^8^, and additive manufacturing materials^9,10^, as well as the structural components^11,12^. Heat fusion or thermal welding of thermoplastics is a common means to join polymeric parts, where two surfaces are brought into close contact above their glass transition temperature, $T_{\mathrm{g}}$, allowing inter-diffusion over a period of time, *t*^13^. The fusion of thermoplastics has drawn special attentions due to the need for high-performance regenerated interfaces in polymers or composites^9,14,15^.

There have been plenty of efforts devoted to understanding microstructural evolution at the thermoplastic interfaces and its correlation with their mechanical performance. In theoretical studies, the diffusive motion of polymer chains is usually assumed to be constrained within the initial tubes^16^. A reptation model was then employed to calculate the growth of fusion interfaces between identical or compatible polymers, yielding a scaling relation between the thickness (*h*) and time of fusion (*t*) before the equilibrium is established^17^, that is, $h(t)\sim t^{1/2}$. This model also predicts the strength of interfaces as a function of *t* and molecular weight (*M*) as ${\sigma }_{\mathrm{s}}\sim t^{1/4}M^{\alpha }$, where $\alpha = -1/4$ within the tube renewal time $T_{\mathrm{r}}$, and $-3/4$ for $T > T_{\mathrm{r}}$^17^. Microscopic dynamics of diffusion and randomization is considered in this model. For interfaces between two immiscible polymers, Helfrand and Tagami^18,19^ estimated the upper limit of the interfacial thickness by following the Flory-Huggins mixing equation, which is proportional to ${\chi }^{-1/2}$. Here $\chi$ is the interaction parameter between the two polymers. The aforementioned theories of fusion are limited to the amorphous polymers in the melt state or above glass transition temperature, $T_{\mathrm{g}}$. As shown by Boiko et al.^20^, the theory developed for amorphous polymers do not apply for the semi-amorphous ones in the vicinity of $T_{\mathrm{g}}$, as in the injection molding^9^ and additive manufacturing of polymers^10^. A multiphysical framework incorporating the processes of heat transfer, mass transport, and crystallization is developed to model the underlying complexity of fusion^21^. However, the molecular-level picture behind the scaling relation, the key kinetic steps such as diffusion and entangling, and the physical significance of $T_{\mathrm{r}}$ are not resolved. The effects of fusion conditions on the mechanical performance of the polymer interfaces are also not well clarified.

Molecular dynamics (MD) simulations identify two different stages of inter-diffusion, where the first stage is faster than the second one^22,23^. This finding can be explained by the existence of unequilibrated chain ends and vacancies on both sides of the interface in the initial stage where diffusive processes dominate^23^. The interfacial confinement on polymers increases as the diffusion proceeds, involving more significant entanglement effects^22^. The fact suggests that inter-diffusion and entanglement could dominate at different kinetic steps. This proposal can also be supported by the recovery of mechanical properties at the interface. While polymer melting dynamics predicts that the polymer chains should diffuse by a distance on the order of their radius of gyration to fully erase the memory of structural information at the interfaces, experimental^24^ and simulation^11,22,23^ studies suggest that the bulk strength can be recovered at much earlier time, due to the established entanglement between polymer chains that controls the interfacial strength and plastic responses^13^.

The entanglement density of polymer chains was measured and associated with the mechanical responses through the specific penetration energy in experimental ballistic tests^25^. Molecular simulations reveal increase in strength with time of fusion in terms of entanglement, which is quantified through the evolution of topological constraints (TCs)^13,26^. The mode of failure for the interfaces changes from pull-out to chain scission with a growing density of TCs. However, the relationship between the dominating microscopic dynamics of polymers in the two key kinetic steps and the mechanical properties such as stiffness and strength has not been well addressed. In practice, multiple factors including the viscosity and compatibility of polymers, the ambient temperature and pressure, as well as the time of fusion control the kinetics of fusion. Rational design of a fusion process beyond the trial-and-error approach may save the cost and improve the performance of the fusion product^27^. Besides, interfacial engineering through the chemistry and structures of polymers could also be beneficial for improved efficiency of fusion^28^, compatibility^29^ and strength of fused interfaces^30^. Therefore, it is necessary to explore the evolution of molecular structures upon the fusion process, and clarify the scaling behaviors of the interfacial properties under specific processing conditions. Optimized solutions to fusion toward functional material design or recycling can be achieved.

**Figure 1:**
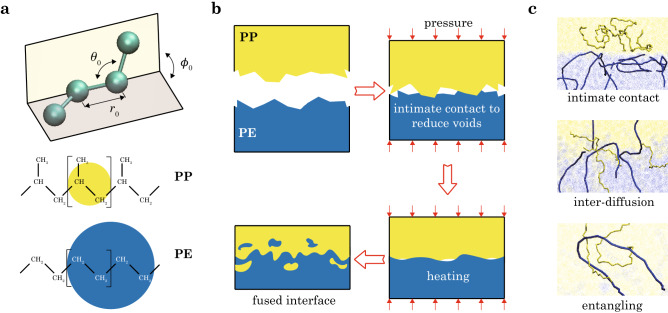
(**a**) Coarse-grained models of PE and PP. (**b**) Physical processes in heat fusion: (**c**), (1) intimate contact, (2) inter-diffusion, and (3) entangling.

To elucidate this processing-microstructures-performance relationship, molecular simulations for the microscopic mechanisms should be combined with experimental characterization of the structural and mechanical properties at the specimen level. In this work, we use coarse-grained (CG) models to extend spatial and temporal spans of the simulation scales. Two widely-used thermoplastic polymers, polyethylene (PE) and polypropylene (PP), are studied with a focus on the characteristic kinetic processes occurring at their heat-fusion interfaces (Fig. 1). Complementary hot-compression-molding (HCM) experiments are carried out to assess the kinetic effects of material microstructures and performance at the specimen level.

## Results

### Kinetic processes in heat-fusion

**Figure 2:**
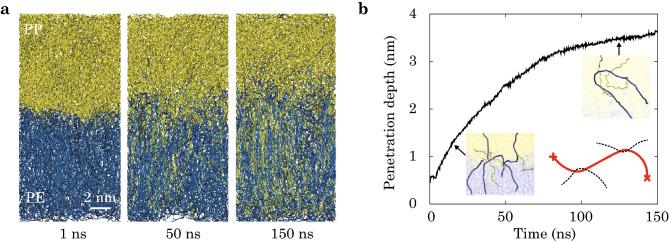
(**a**) Snapshots of heat-fusion processes at the PE (bottom)/PP (upper) interface and (**b**) the penetration depth plotted against fusion time, obtained from the coarse-grained molecular dynamics (CGMD) simulations. The insets of representative polymer chains illustrate kinetic steps of inter-diffusion and entangling, respectively. A cartoon showing the definition of two topological constraints (TCs, black) on a primitive path (red) is also included. The fusion conditions are 500 K and 1 atm.

After intimate contact, the polymer chains penetrate the PE/PP interfaces during heat fusion (Fig. 2a). The depth of penetration is defined as $h = 1/N \sum |z_i - z_0|$, where *N* is the number of beads that cross the interface, $z_0$ is the position of interface, and $z_i$ is the position of *i*-th bead. The penetration depth increases with time of fusion as $h \sim t^{\gamma }$ (Fig. 2b), where the exponent ${\gamma } = 0.52$ is close to the value predicted from the reptation model (0.5)^17^. In the beginning of fusion processes, PE and PP are forced to make intimate contact. By identifying the morphologies of the polymer chains, we can divide the heat-fusion processes into two kinetic steps after intimate contact, which are dominated by (1) inter-diffusion and (2) entangling, respectively (Fig. 1c). The non-zero value of penetration depth at the beginning of fusion originates from intimate contact (Fig. 2b). The process is followed by inter-diffusion of polymer chains, crossing the interface first and then mixing transversely. These features are illustrated in the insets of Fig. 2b, where the chains intercalate the opposite bulk polymer nearly normal to the interface at the early stage, resulting in quick increase in the penetration depth. In the subsequent process of fusion, entanglement between chains from both sides of the interface serves as TCs to the inter-diffusion. The increase in penetration depth is then slowed down as entanglement is initiated.

The CG models use much shorter polymer chains ($\sim 10^4$ g/mol) than those in experimental samples ($\sim 10^5{-}10^6$ g/mol)^31^, although the entanglement effect is included. The size effects on the chain dynamics have to be addressed. To assess the local order in polymer chains, we define a parameter $s_i = (3\langle \cos ^2 {\theta }_{ij} \rangle - 1)/2$ for the *i*-th triplet on the chains, where ${\theta }_{ij}$ is the angle between the *i*-th triplet and its neighbor, the *j*-th triplet^32^. The order parameter of a chain is evaluated as the site average, that is, $S = \sum _{i=1}^{N}{s_i}/N$, where *N* is the number of triplets in the chain. $S = 0$ and 1 corresponds to the disordered and ordered limits. Structural evolution of PE with two chain lengths, *n*50 and *n*100, is summarized in Fig. S1. The model with very short chains (*n*50) relaxes quickly into highly ordered structures with $S = 0.9$. The time scale of self-arrangement for them is equal or shorter than that for inter-diffusion, which can have strong impact on the polymer dynamics but not relevant for samples with long chains. Consequently, our following discussions are focused on the models of *n*100 and *n*200.

### Microstructural evolution

**Figure 3:**
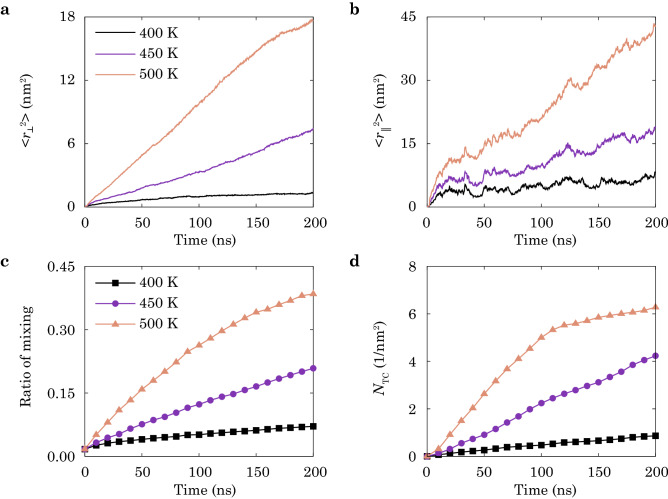
CGMD simulation results of the microstructural indicators of the PE/PP heat-fusion processes at different fusion temperature, $T = 400, 450, 500$ K. (**a**,**b**) Mean-square distances (MSDs) of polymer chains across the interface, where $\langle r_{\Vert }^2 \rangle$ is the component parallel to the interface, and $\langle r_{\bot }^2 \rangle$ is the component normal to the interface. (**c**) Ratio of mixing and (**d**) areal density of TCs, $N_{\mathrm{TC}}$, are calculated against the time of fusion.

To quantitatively assess microstructural evolution during interfacial heat fusion, a few descriptors are defined to characterize the kinetic steps. We first calculate the mean square distances (MSDs) of the chains that cross the interface. The normal ($\langle r^2_{\bot } \rangle$) and parallel ($\langle r^2_{\Vert } \rangle$) components measured to the interfaces are plotted in Fig. 3a,b. The results show that $\langle r^2_{\bot } \rangle$ grows fast during the initial stage. In contrast, $\langle r^2_{\Vert } \rangle$ shows mild increase with decreasing rate at long fusion time, implying the transition in kinetic steps from crossing the interface to transverse mixing. Elevating temperature leads to large values of MSDs regardless of their directions.

The ratio of mixing is used to measure the degree of mixing at the interfaces, which is calculated as $m = \sum ^n_i P_i / \sum ^n_i M_i$. Here *n* is the total number of beads, $P_i$ is the number of PE-PP pairs for the *i*-th bead, and $M_i$ is the number of total pairs for the *i*-th bead. The pairs of beads are defined as the two beads within a cut-off distance, $r_{\mathrm{c}}$ (Table 1). The ratio of mixing is plotted against fusion time in Fig. 3c, which is small at low temperature (400 K), indicating a thin and poorly-fused interfacial layer. Further decreasing the temperature leads to a complete non-fused interface.

The entanglement between chains from the polymer pairs are regarded as TCs, which are calculated following the primitive path analysis^26^. In this method, each polymer chain is considered to be staying within a primitive path, which is obtained by contraction while keeping the ends of chains fixed. As illustrated in the inset of Fig. 2b, contacts between the resulting primitive paths are counted to determine the number of TCs^26^. The areal density of TCs, $N_{\mathrm{TC}}$, are plotted against fusion time in Fig. 3d. The results show that the formation of entanglement slows down the penetration (Fig. 2b) and mixing (Fig. 3c) processes, suggesting that the inter-diffusion is impeded by entanglement.

**Figure 4:**
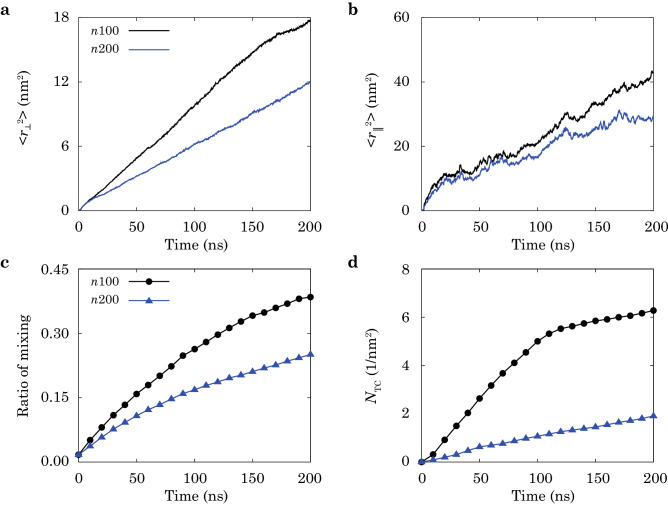
CGMD simulation results of the microstructural indicators measured for the PE/PP heat-fusion processes of polymer chains with different lengths, *n*100 and *n*200. (**a**,**b**) Mean-square distances (MSDs) of polymer chains across the interface, where $\langle r_{\Vert }^2 \rangle$ is the component parallel to the interface, and $\langle r_{\bot }^2 \rangle$ is the component normal to the interface. (**c**) Ratio of mixing and (**d**) areal density of TCs, $N_{\mathrm{TC}}$, are calculated against the time of fusion. The fusion conditions are 500 K and 1 atm.

The effects of viscosity are assessed by using models with different molecular weights or chain lengths. As presented in Fig. 4a,b, shorter chains (*n*100) diffuse at larger distances in both the directions normal and parallel to the interface. The zero-rate shear viscosity ${\eta }_0$ of polymer chains in the melt scales with the molecular weight *M* in a power law, ${\eta }_0 = KM^{\alpha }$, where *K* and $\alpha$ are parameters for given polymers^33,34^. For *M* lower than a certain threshold value $M_{\mathrm{c}}$, entanglement between chains can hardly be established, and ${\eta }_0$ is proportional to *M* ($\alpha = 1$). Above $M_{\mathrm{c}}$, the exponent is $\alpha \approx 3.4$. From the entanglement identified in simulations, this relation suggests that the value of ${\eta }_0$ for the *n*200 model is nearly ten times higher than that of *n*100, indicating a much smaller ratio of mixing (Fig. 4c) and $N_{\mathrm{TC}}$ (Fig. 4d) that is consistent with the results in Fig. 3.

**Table 1:** Parameters of coarse-grained models for PE and PP^43^.

Parameters	Values
Spring constant of PE, $k_{\mathrm{s}}$	4.78 ${\mathrm{kcal}}/({\mathrm{mol}}^{2})$
Spring constant of PP, $k_{\mathrm{s}}$	114.8 ${\mathrm{kcal}}/({\mathrm{mol}}^{2})$
Equilibrium distance of PE, $r_{0}$	0.46 nm
Equilibrium distance of PP, $r_{0}$	0.298 nm
Fracture strain of bonds for PE and PP, $\varepsilon _{\mathrm{f}}$	20%
Angle constant of PE, $k_{\mathrm{b}}$	5.98 kcal/mol
Angle constant of PP, $k_{\mathrm{b}}$	22.1 kcal/mol
Equilibrium angle of PE, ${\theta }_0$	180°
Equilibrium angle of PP, ${\theta }_0$	117°
Dihedral parameters of PE, $C_1, C_2, C_3, C_4, C_5$	− 4.699, 0.361, 0.0406, − 0.0105, − 0.00048 kcal/mol
Dihedral parameters of PP, $k_{\phi 1}, {\phi }_1, k_{\phi 2}, {\phi }_2$	0.741 kcal/mol, 100°, − 1.410 kcal/mol, 190°
Lennard-Jones 12-6 parameters of PE, ${\epsilon }, {\sigma }$	0.84 kcal/mol, 0.47 nm
Lennard-Jones 12-6 parameters of PP, ${\epsilon }, {\sigma }$	0.63 kcal/mol, 0.43 nm
Cut-off distance for Lennard-Jones 12-6 for PE and PP, $r_{\mathrm{c}}$	1.1 nm

### Mechanical performance of heat-fusion interfaces

**Figure 5:**
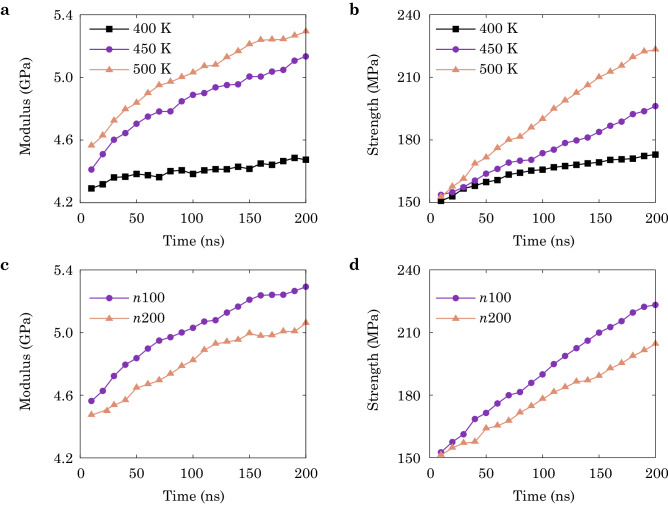
(**a**) Young’s modulus and (**b**) the tensile strength of the fused PE/PP interfaces plotted against the fusion time at different temperature ($T = 400, 450, 500~$K) in the CGMD simulations. (**c**) Young’s modulus and (**d**) the tensile strength of fused PE/PP interfaces plotted against the fusion time with different chain lengths, *n*100 and *n*200 in the CGMD simulations. The temperature is controlled at 500 K for data shown in (**c**) and (**d**).

The mechanical performance of the heat-fusion interfaces are tested under uniaxial tension. In the CGMD simulations, the Young’s modulus of the PE/PP interfaces is measured from the linear part of stress–strain curves and plotted against the fusion time *t* as shown in Fig. 5a,c. The stiffness of the interface increases with *t* and temperature *T* (Fig. 5a). Similar as the microstructural descriptors, the Young’s modulus shows nonlinear dependence on *t*. Fitting the data yields a scaling relation of $t^{{\beta }}$, which is $t^{0.013},~t^{0.050},~t^{0.054}$ for $T = 400,~450,~500~$K, respectively. Although the scaling exponents of modulus are much smaller than those estimated from the penetration depth $(t^{0.145},~t^{0.372},~t^{0.435})$ (Fig. S2a) and ratio of mixing $(t^{0.345},~t^{0.653},~t^{0.691})$ (Fig. 3a), the trend of increasing signals the correlation between microstructural evolution during fusion and mechanical performance of the product.

The yield strength of PE/PP interfaces is also measured and plotted in Fig. 5b,d. Compared with the Young’s modulus, the strength converges slower than the modulus. Fitting strength-time data with the scaling relation $t^{\beta }$ yields $t^{0.048}$, $t^{0.087}$, $t^{0.141}$ for $T = 400,~450,~500~$K, respectively. It is noted that, samples fused at 450 and 500 K have close exponents in the Young’s modulus ($t^{0.050}$, $t^{0.054}$), while the samples fused at 500 K ($t^{0.141}$) show larger exponents for strength than samples fused at 450 K ($t^{0.087}$). All strength exponents are greater than those of the modulus at the same conditions, suggesting stronger dependence on the fusion time. This can be explained by the fact that the modulus is the results of average statistics, while the strength subjects to extreme statistics. At the kinetic step of inter-diffusion, the Young’s modulus increases with the overlap between chains, which saturates before the completion of entanglement. However, because of the low density of TCs, chain sliding normal to the interface results in a low strength. As the TCs are enhanced with longer fusion time, the strength is largely improved, while the increased TCs have a minor effect on modulus. This contrast in the *t*-dependence of Young’s modulus and tensile strength was also reported in previous studies^22,23^. The effects of viscosity are shown in the results with different chain lengths (Fig. 5c,d). Shorter chains (*n*100) with a higher mobility demonstrate larger modulus and higher strength than long chains (*n*200), aligning well with the findings from microstructural evolution (Fig. 4). Fitting the scaling relation yields $t^{0.054},~t^{0.048}$ for the modulus and $t^{0.141},~t^{0.109}$ for the strength, for *n*100 and *n*200, respectively. These results also agree with the scaling relations for the penetration depth $(t^{0.435},~t^{0.368})$ and ratio of mixing $(t^{0.691},~t^{0.635})$.

High contrast in the microstructural evolution of fused interfaces is identified under uniaxial tension (Figs. S3 and S4). To characterize the load distribution in the samples, we introduce the spatial load filling factor^35^, $q_{\mathrm{f}} = D/l$. Here $D = 1/\sum _{i = 1}^l I_i^2$ is the participation ratio and $I_i \left( \sum _{i = 1}^l I_i = 1\right)$ is the normalized load on *i*-th chain $(i = 1, 2, 3, ..., l)$ distributed within the cross-section. Therefore, $q_{\mathrm{f}}$ varies from 0 and 1 for concentrated and uniformly distributed tension, respectively. For poorly-fused interfaces ($t = 10$ ns), $q_{\mathrm{f}}$ is nearly a constant in the bulk region of PE and PP at small strain ($\varepsilon = 1~\%$, Fig. S3a). The PP part has a higher value of $q_{\mathrm{f}}$ than PE, indicating better load transfer as PP has a higher strength. As strain increases (Fig. S3b,c), $q_{\mathrm{f}}$ in PP increases, suggesting improved loading distribution, while $q_{\mathrm{f}}$ in PE decreases dramatically at large strain (${\varepsilon = 20~\%}$), accompanied by a large number of fractured bonds. The load is here restricted by fracture of PE chains, and the stress declines as shown in the stress–strain curves (Fig. S2c). For well-fused interfaces ($t = 200$ ns), $q_{\mathrm{f}}$ is higher in the mixed interface than that in pure PE at small strain (Figs. S3a and S4a), which results in a higher Young’s modulus as presented in Fig. 5a,c. Since the load transfer is enhanced by TCs (Fig. S4b,c), $q_{\mathrm{f}}$ of the well-fused interface ($t = 200$ ns) is larger than that of the poorly-fused interface ($t = 10$ ns). The energy can be dissipated by sliding and disentangling of the chains, and the ductile behaviors are identified by the stress–strain curves in Fig. S2c,d.

### Experimental results

CGMD simulation results outline the key kinetic steps during heat fusion, as well as their effects on the evolution of interfacial microstructures and mechanical performance at a molecular level. However, heat fusion is a multi-scale process in space and time. Although the findings are expected to be valid at the material level, slower processes at larger length scales such as heat diffusion and non-uniform deformation should be included for additional discussion. However, modeling across these scales are challenging. Consequently, we conduct experiments to validate our arguments and gain further insights into the heat-fusion processes. The focus will be placed on validating the two-step nature of the fusion kinetics, and additional effects on the mechanical properties of the fused interfaces that cannot be included in the simulations, such as the viscosity, compatibility and crystallization. Two types of PE are considered. The linear-low-density PE (LLDPE) has a higher compatibility with PP than the high-density PE (HDPE). To inspect the effect of viscosity, two types of PP are used. PP-2 has a relatively lower melting flow index (MFI = 0.5 g/10 min) than that of PP-1 (MFI = 12 g/10 min). MFI is a measure for the ease of flow of melted thermoplastics, a lower value of which indicates a higher viscosity.

**Figure 6:**
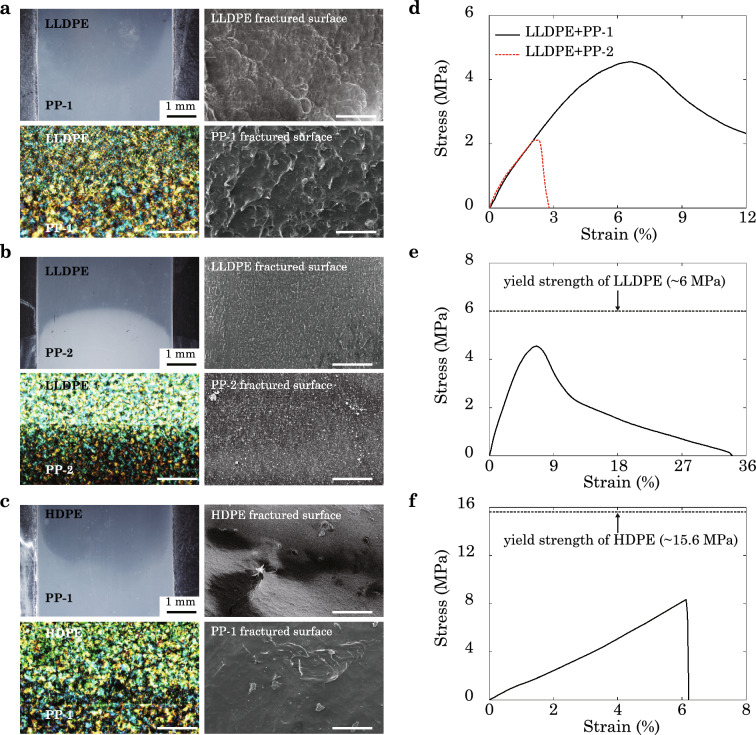
Experimental characterization of (**a**) LLDPE/PP-1, (**b**) LLDPE/PP-2, and (**c**) HDPE/PP-1 interfaces, where the left top panel is obtained from optical microscope (OM), the left bottom is from polarized light microscope (PLM), and the right two panels are from scanning electron microscopy (SEM). The unlabelled scale bars are 100 µm. (**d**–**f**) Experimental results of the typical stress–strain curves of LLDPE/PP-1 and LLDPE/PP-2 (**d**), LLDPE/PP-1 (**e**) and HDPE/PP-1 (**f**) samples fabricated by HCM. All the samples are fused for 1 min.

We use HCM to manufacture PE/PP samples under different processing conditions (Fig. S5, see details in “Methods”). Among the four types of pairs, the best fused PE/PP pair is LLDPE/PP-1 (Fig. 6a) according to the visual characterization by the optical microscope (OM) and polarized light microscope (PLM). In contrast, the pair of LLDPE and PP-2 shows a distinct interface. The samples are then tested by uniaxial tension with a fixed gauge length, $l_{\mathrm{g}} = 30$ mm, and the relative displacement of clamps is recorded as well as the tensile force. Typical stress–strain curves of bulk and fused samples are obtained (Figs. S6 and S7). Poorly-fused interfaces result in weak mechanical performance. As shown in Fig. 6d, the LLDPE/PP-1 sample outperforms LLDPE/PP-2 in both strength and strain to failure. Evidenced from the scanning electron microscopy (SEM) images (Fig. 6a,b), LLDPE/PP-1 samples present rough fractured surfaces from both PE and PP sides, implying pull-out and fracture of polymer chains, while the LLDPE/PP-2 samples have much smoother surfaces. It is also noted that, the Young’s moduli of LLDPE/PP-1 and LLDPE/PP-2 are quite close, although their interfaces differ greatly in fusion (Fig. 6a,b). Similar behaviors are found in our simulation results, where the morphological characterization (Figs. S3a and S4a) shows high contrast between poorly- and well-fused interfaces, although their Young’s moduli are close (Fig. S2c,d). These results suggest that, inter-diffusion, as the first kinetic step, improves the Young’s modulus by establishing overlap between chains across the interface. In the second kinetic step, the entanglement of chains is achieved through a much slower process, especially for long chains with high viscosity (Fig. 4d). Mechanical properties associated with entanglement such as tensile strength and plastic responses require more time to recover their bulk limits than that for the modulus. Experimental results thus further validate that the dependence of modulus on the degree of fused interface or fusion time is less than that of the strength.

Comparability between thermoplastics is another key factor that modulates the heat fusion process. According to the Flory-Huggins equation, the free energy of mixing is $F_{\mathrm{m}} = \frac{RTV}{V_{\mathrm{R}}}({\varphi }_{\mathrm{A}} x_{\mathrm{A}} \ln {\varphi }_\mathrm{A} + {\varphi }_{\mathrm{B}} x_{\mathrm{B}} \ln {\varphi }_{\mathrm{B}} + {\chi }{\varphi }_{\mathrm{A}}{\varphi }_{\mathrm{B}})$, where $V_{\mathrm{R}}$ is the referenced volume, ${\varphi }$ is volume fraction, *x* is the degree of polymerization, and $\chi$ is the interaction parameter. A higher value of $\chi$ means lower compatibility or miscibility. The value of $\chi$ for specific pair can be calculated as ${\chi }_{\mathrm{A-B}} = \frac{V_{\mathrm{R}}}{RT}({\partial }_{\mathrm{A}} - {\partial }_{\mathrm{B}})^2$, where ${\partial }$ is the solubility parameter estimated from the contributions of functional groups^36^. We thus have ${\chi }_{\mathrm{LLDPE/PP-1}} = 0.062$ and ${\chi }_\mathrm{HDPE/PP-1} = 0.18$. Although LLDPE and HDPE have close values of MFI, 1.0 and 2.2 g/10 min, the degree of fused interfaces is largely restricted by their compatibility. The higher value of ${\chi }_\mathrm{HDPE/PP-1} = 0.18$ results in poorer compatibility of the HDPE/PP-1 pair than that of LLDPE/PP-1 (${\chi }_{\mathrm{LLDPE/PP-1}} = 0.062$), which leads to poorly-fused interface as identified in Fig. 6c.

As processed in the same conditions, the strength reduction compared to the pristine samples is more significant for low-compatibility interfaces (Fig. 6e,f). The LLDPE/PP-1 sample reserves $\sim 80\%$ of the strength after fusion, while the HDPE/PP-1 sample has only half of the strength in the condition $T = 200$ °C. The poorly fused interfaces also suffer from low strain to failure. The stress–strain curve of HDPE/PP-1 shows brittle behaviors  and their fractured surfaces are smoother than those of LLDPE/PP-1 (Fig. 6a,c).

**Figure 7:**
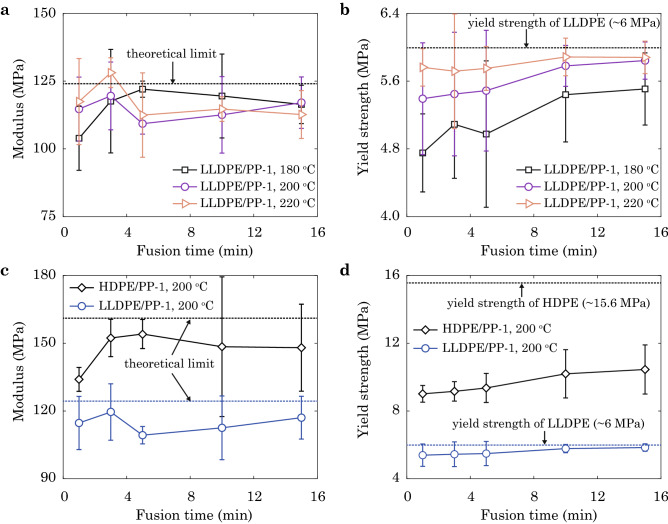
(**a**) Experimental results for the Young’s modulus and (**b**) yield strength of LLDPE/PP-1 samples obtained at $T = 180,~200,~220~\mathrm{^{\circ }C}$ in HCM. (**c**,**d**) Experimental results for LLDPE/PP-1 and HDPE/PP-1 samples obtained at $T = 200~\mathrm{^{\circ }C}$. Theoretical limits of moduli obtained from the rule of mixture and strengths of pure samples are plotted as dash lines.

The Young’s modulus and yield strength of LLDPE/PP-1 samples processed at different temperature and time of fusion are summarized in Fig. 7a,b. A theoretical limit of modulus can be calculated from the rule of mixture $Y = 2/(Y_{\mathrm{PE}}^{-1} + Y_\mathrm{PP}^{-1})$ as $Y_{\mathrm{LLDPE/PP-1}} = 124.9$ MPa, using parameters measured from pure samples in uniaxial tensile tests, $Y_{\mathrm{LLDPE}} = 97.9$ MPa, $Y_{\mathrm{HDPE}} = 134.8$ MPa, and $Y_{\mathrm{PP-1}} = 202.7$ MPa. The moduli measured for our fused samples do not show notable time dependence for *t* ranging from 1 to 15 mins, and the values for samples obtained at 180, 200 and $220~\mathrm{^{\circ }C}$ are all close to the theoretical limit, indicating that the first step of inter-diffusion is fast. In contrast to the modulus, the strength increases with *t*, scaling as $t^{0.055},~t^{0.032},~t^{0.009}$ for $T = 180$, 200 and $220~\mathrm{^{\circ }C}$, respectively, which clearly demonstrates the two-step nature of the fusion kinetics and echoes the findings in molecular simulations. It should be noted that, the exponents reported from the experiments decrease with the temperature while the exponents from the simulation results $(t^{0.048},~t^{0.087},~t^{0.141})$ increase with the temperature in a different range of temperature $(400, 450, 500~\mathrm{K})$. This disagreement is attributed to the fact that different time scales are used to fit the exponents in the experiments and simulations (Figs. 5 and 7). As the time of fusion increases, the interface is strong, and thus the strength is dominated by the part of bulk LLDPE (Fig. 7b). Samples fused at $220$ °C reaches $\sim ~95\%$ of the pristine strength of LLDPE for 1 min, while samples fused at $180$ °C need 10 mins for $\sim ~90\%$ recovery, which demonstrates the effect of temperature on accelerating the interfacial fusion. Well-fused interfaces stiffen after yielding (Fig. S7). The interfaces can be stronger than bulk LLDPE, and thus the LLDPE part is stretched to large strain with necking. Comparing the results in Figs. 7b and S8a, the failure strength is higher than the yield strength for LLDPE/PP-1 samples fused at $220$ °C due to this stiffening behavior. The ductility of all samples is summarized in Fig. S8b. For samples fused at $220$ °C or lower temperature with longer fusion time, the failure strain can be greater than the yield strain ($\sim 10\%$) by one order of magnitude, suggesting outstanding ductile performance (Fig. S8b). It is noted that samples fused at 200 and 220 °C for 10 min have close yield strengths (5.78 and 5.88 MPa in Fig. 7b). However, the strain to failure of the latter sample $(215 \%)$ is nearly twice of the value for the former one $(124 \%)$. This discrepancy in strain to failure reveals the hidden information in the microstructures, such as the density and distribution of the TCs across the interface.

The effect of compatibility is demonstrated in Fig. 7c,d. The moduli of LLDPE/PP-1 and HDPE/PP-1 samples show negligible *t*-dependence except for the HDPE/PP-1 sample fused for a short duration of 1 min. Although the moduli measured for LLDPE/PP-1 and HDPE/PP-1 samples are close to the theoretical limits ($Y_\mathrm{LLDPE/PP-1} = 124.9$ MPa and $Y_{\mathrm{HDPE/PP-1}} = 162.0$ MPa), the contrasts in yield strength and failure strain are significant. LLDPE/PP-1 is more compatible than HDPE/PP-1, and thus approaches the pristine limit within 15 min, while the latter sample gains only $\sim 67\%$ of the limit at the same time. In contrast to the ductile behaviors of LLDPE/PP-1, HDPE/PP-1 samples fail by brittle fracture (Fig. 6c), with failure strain below $10\%$ and much lower than that of LLDPE/PP-1 (Fig. S8b).

## Discussion

The approach combining molecular-level simulations and structural-level experiments in this study is to elucidate the underlying molecular mechanisms of the processing-microstructures-performance relationship of heat-fused thermoplastic polymers, while including the processes occurring at spatial and temporal scales beyond the capability of MD simulations, even in a CG representation. Our experimental data is in general consistency with the CGMD simulation results, though the specific values such as Young’s modulus and tensile strength are not in quantitative agreement due to the mismatch in the length and time scales of fusion, the material imperfections in experimental samples, and the relatively high loading rate in simulations. Given the characteristics of microstructural evolution obtained from simulations, we find that the Young’s modulus and strength show different dependence on the time of fusion (Fig. 5), which is explained from the two kinetic steps captured from the structural descriptors (inter-diffusion and entanglement, see Figs. 3 and  4). The modulus is dominated by the first step, while the strength and ductile behaviors are more sensitive to the entangling process in the second one. The experimental data support this argument by showing the difference between well- and poorly-fused interfaces of LLDPE/PP (Fig. 6a,b,d). Since the first kinetic step is established at a shorter time scale than the second one^22,23^, the modulus converges faster than the strength, which is validated by our experimental results for the Young’s modulus and yield strength (Fig. 7a,b). The CGMD simulation results are in accord with those reported from the experiments by giving the larger strength-time exponents $$(t^{0.048}$$, $$t^{0.087}$$, $$t^{0.141})$$ than the modulus-time relations $$(t^{0.013},~t^{0.050},~t^{0.054})$$. To assess the quality of fused interfaces beyond the recovery of strength, the ductile behaviors are extracted, which also demonstrate the significance of TCs (Figs. 7b and S8). The disentanglement and sliding of chains result in additional energy dissipation, and improve the load transfer as demonstrated by the CGMD results (Figs. S2–S4).

Crystallization is another important process during fusion, which unfortunately cannot be well captured by the CG approach adopted here^37,38^. Interfacial entanglement established in the melt state could be anchored in chain-folded lamellae upon crystallization by using metallocene-catalyzed polymers^39^. However, our HCM samples are obtained from conventional heterogeneous Ziegler-Natta reactions, where the accumulation of amorphous polymers at the fusion interface is expected to decouple interfacial entanglement and reduce the strength (Fig. 7b,d). The choice of Ziegler-Natta polymers is thus compatible with the condition of simulations, where the slow process of crystallization is not fully captured for limitation in the time scale. On the other hand, the presence of an interface, such as that between the fibers and polymer matrices in a composite, can nucleate crystallization^40^. Our in-situ observation of the isothermal crystallization at LLDPE/PP-1 interfaces, however, suggest that the crystallization of LLDPE and PP-1 starts separately, and no interface-induced nucleation is found (Fig. S9). Hence, we conclude that the crystallization has minor effects on the degree of fusion in our samples.

In our experimental results, instead of direct characterization of the kinetics at the fused interfaces, the evolution of their mechanical responses is discussed (Figs. 7 and S8). To fill the gap between these results and the molecular dynamics reported from the simulations, advanced techniques such as the charge dependence of three-dimensional atomic force microscope (3D AFM)^41^ and neutron reflectivity (NR)^42^ may be used to resolve macromolecular diffusion and entanglement. On the other hand, in contrast to the fusion by HCM, the process of injection is more complicated for the coupling between the processes of momentum, heat, and mass transfer^10^. Due to the decrease of temperature during injection, the inter-diffusion and entangling processes are accompanied by crystallization. Further exploration to elucidate the underlying physical and microstructural complexities could offer more insights into the fused polymer interfaces from the processes of injection molding^9,21^ and material-extrusion in polymer additive manufacturing^10^.

## Conclusion

We demonstrated, at the molecular and structural level, two key kinetic steps (inter-diffusion and entanglement) in the heat-fusion process of thermoplastic PE/PP interfaces. The features of these processes are characterized by microstructural evolution of the polymer chains, which define the network structure of polymer chains and mechanical performance of heat-fusion interfaces. Mechanical properties of heat-fusion interfaces such as the Young’s modulus and tensile strength show different sensitivities to these two kinetic steps. The Young’s modulus usually recovers faster than the strength that is controlled by polymer entanglement to a large extend, rather than diffusion. The heat-fusion processes can thus be designed by controlling these steps by, for example, increasing the processing temperature, reducing the viscosity of polymer, or choosing pairs with high compatibility, and extending fusion time, to promote the performance of thermoplastic interfaces and composites in engineering. The current work combining molecular-level simulations and structural-level experiments presents quantitative discussion on the underlying processing-microstructures-performance relationship.

## Methods

### Coarse-grained models and molecular dynamics simulations

The atoms on PE and PP chains are grouped into clusters and represented by the CG beads following the scheme developed in the MARTINI force field^43^. For PE, two monomers are mapped into a CG bead, while in PP, each monomer is mapped into one CG bead (Fig. 1a). Stretching between adjacent beads at a distance of *r* is described by harmonic bonds with energy $E_\mathrm{s} = k_{\mathrm{s}}(r-r_{0})^2$, where $r_0$ is the equilibrium distance and $k_{\mathrm{s}}$ is the spring constant.

A continuous triplet of beads is subjected to an energy term of angle interaction ($\theta$), represented by harmonic cosine functions, $E_{\mathrm{b}} = k_{\mathrm{b}}({\theta } - {\theta }_0)^2$, where ${\theta }_0$ is the equilibrium angle and $k_{\mathrm{b}}$ is the stiffness parameter. It was reported that the CG model of PE chains generates numerical instabilities and should be corrected by using the RB function^44^, while for PP, conventional dihedral terms are sufficient^43^. Consequently, PE dihedrals are described by the Rychaert-Bellemans (RB) function^43^, $E_{\mathrm{d}} = \sum _{i = 1}^5 C_i {\cos }^{i-1}{\phi }$, and PP dihedrals by a sum of two proper dihedral functions, $E_{\mathrm{d}} = \sum _{i = 1}^2 k_{\phi i}\left[ 1 + \mathrm{cos}({\phi } - {\phi }_i)\right]$. Here $C_{i}$, $k_{\phi i}$, ${\phi }_i$ are dihedral parameters. The non-bonding interaction is described through the Lennard-Jones 12-6 potential function, $E_\mathrm{LJ} = 4{\varepsilon }\left[ (\frac{\sigma }{r})^{12} - (\frac{\sigma }{r})^{6} \right]$, where $\varepsilon$ and $2^{\frac{1}{6}}\sigma$ are the binding energy and equilibrium distance, respectively. Parameters used in the CG models are summarized in Table 1.

The initial configurations of polymer chains are generated for PE and PP by using the Moltemplate code^45^. The lengths of single chains are chosen within the range of $n = 50{-}200$ beads for PE, and $65{-}260$ beads for PP, corresponding to a molecular weight up to 11, 200 g/mol. The choice of *n* is made to save computational costs. The entanglement molecular weight of PE and PP are $1200{-}2100$ and $2800{-}7100$ g/mol, respectively. As a result, the entanglement effect can be explored. The pairs of PE and PP are controlled to have nearly the same molecular weight. Specifically, three models of PE and PP are constructed in this work: (1) *n*50 represents 50 PE beads or 65 PP beads; (2) *n*100 represents 100 PE beads or 130 PP beads; (3) *n*200 represents 200 PE beads or 260 PP beads. The dataset of *n*100 is used for discussion if not specified. The generated networks of chains are relaxed at relatively high temperature (600 K, 1 atm) for a long period of 50 ns, and subsequently equilibrated at 1 atm and 400 K, close to the glass-transition temperature ($T_{\mathrm{g}} = 400$ K for PE, 420 K for PP, Fig. S10). The models are then prepared for heat fusion, where PE and PP parts are kept intimate contact with each other under specific conditions of temperature, pressure, and fusion time. Here three replicated models are carried out in the same conditions. All CGMD simulations are performed by using the Large-scale Atomic/Molecular Massively Parallel Simulator (LAMMPS)^46^. The time step to integrate Newtonian equations of motion is 2 fs. Langevin thermostat and Berendsen barostat are used for temperature and pressure control, with damping-time constants of 1 and 100 ps, respectively. In tensile tests, one of the ends of 2 nm in the test specimens (20 nm) are constrained and pulled apart at a loading rate of $5 \times 10^{-2}$ ns$^{-1}$, and the temperature is kept at 300 K.

### Hot-compression molding experiments

Experimental samples of PE and PP are manufactured by HCM (Plate Vulcanizing Machine, Jiehe JH-200T), and the flow chart of fusion is presented in Fig. S5. Here we consider two types of PE, linear-low-density PE (LLDPE) and high-density PE (HDPE). The former has a higher compatibility with PP than the latter. To inspect the effect of viscosity, we use two types of PP. PP-1 has a higher melting flow index (MFI = 12 g/10 min) than that of PP-2 ($=0.5$ g/10 min). LLDPE and HDPE have close MFIs of 1.0 and 2.2 g/10 min. PE and PP used in this work are polymerized by Ziegler-Natta catalysts, and PP is isotactic. We first prepare pure PE and PP samples, by feeding granular raw materials of PE or PP. After molding for 10 min at 200 °C and 10 atm, pure PE and PP samples are obtained. To prepare fused PE/PP interfaces, the pristine samples are cut in half and the pairs of PE/PP halves are replaced in the mold at specific conditions of temperature ($180, 200, 220$ °C), pressure (10 atm), and time of fusion (1, 3, 5, 10, 15 min). Four replicated samples are prepared and tested in the same conditions. The physical processes of heat fusion are illustrated in Fig. 1b,c. The morphologies of PE/PP interfaces are characterized by optical microscope (OM, HIROX MXB-2500REZ) and polarized light microscope (PLM, Olympus BX41P).

Mechanical properties of the fused samples are tested under uniaxial tension (Universal Testing Machine, JinJian UTM-1432). The gauge length is set as the fixed value of $l_{\mathrm{g}} = 30$ mm, and the relative displacement of clamps is recorded as well as the tensile force. Fractography is conducted under the scanning electron microscope (SEM, TESCAN VEGA3). It should be noted that the Young’s modulus and tensile strength of the samples are measured for the whole specimen, which is a composite of the bulk polymer parts and the interface. However, the Young’s modulus is measured at very small strain, the values reported for the composite thus can effectively measure the stiffness of the fusion interface. Moreover, as the samples with reduced mechanical performance fracture in the fused region, the tensile strength of the fusion interface is well characterized. The same arguments apply for the molecular simulations.

## Supplementary Information

Supplementary Figures.

## Data Availability

All data generated or analysed during this study are included in this published article and its supplementary information files.
