# Supplementary material for: Two-step heat fusion kinetics and mechanical performance of thermoplastic interfaces
Source: Sci Rep. 2022 Apr 5;12:5701. doi: 10.1038/s41598-022-09573-3 (PMC8983657; doi:10.1038/s41598-022-09573-3)
Supplement: Supplementary file 1 — Supplementary Figures. [file 41598_2022_9573_MOESM1_ESM.pdf]

Supplementary Information for Two-Step Heat Fusion Kinetics and Mechanical Performance of Thermoplastic Interfaces

Shijun Wang[1,4], Jiaxin Shi[2], Takayuki Shimizu[3,*], Jun Xu[2,*], and Zhiping Xu[1,*]

[1]Applied Mechanics Laboratory, Department of Engineering Mechanics and Center for Nano and Micro Mechanics, Tsinghua University, Beijing, 100084, China
[2]Department of Chemical Engineering, Tsinghua University, Beijing, 100084, China
[3]Strength Research Department, Research & Innovation Center, Mitsubishi Heavy Industries Ltd., Nagoya, 455-8515, Japan
[4]National Center for Nanoscience and Technology, Beijing, 100190, China
[*]Correspondence and requests for materials should be addressed to T. Shimizu (takayuki.shimizu.s2@mhi.com), J. Xu (jun-xu@tsinghua.edu.cn), Z. Xu (xuzp@tsinghua.edu.cn)

ABSTRACT

Supporting Information Available

This Supplementary Information Material includes **Supplementary Figures S1-S10**.

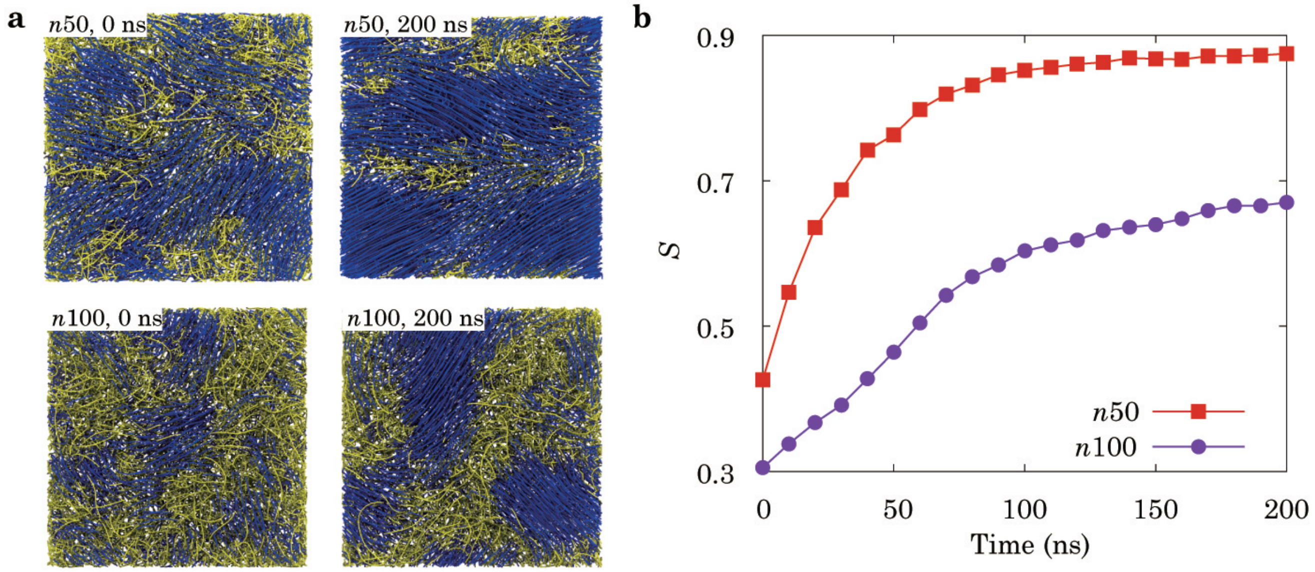

Figure S1. (**a**) Microstructural evolution of PE chains obtained from the CGMD simulations. The ordered region $(s_i > 0.7)$ is highlighted in blue color, and amorphous region $(s_i < 0.7)$ in yellow. (**b**) Self-arrangement of PE chains characterized by the order parameter $S \in [0, 1]$ from the CGMD simulations as a function of fusion time, where the chain length is chosen according to the $n50$ and $n100$ models. Higher values of S indicate more ordered structures.

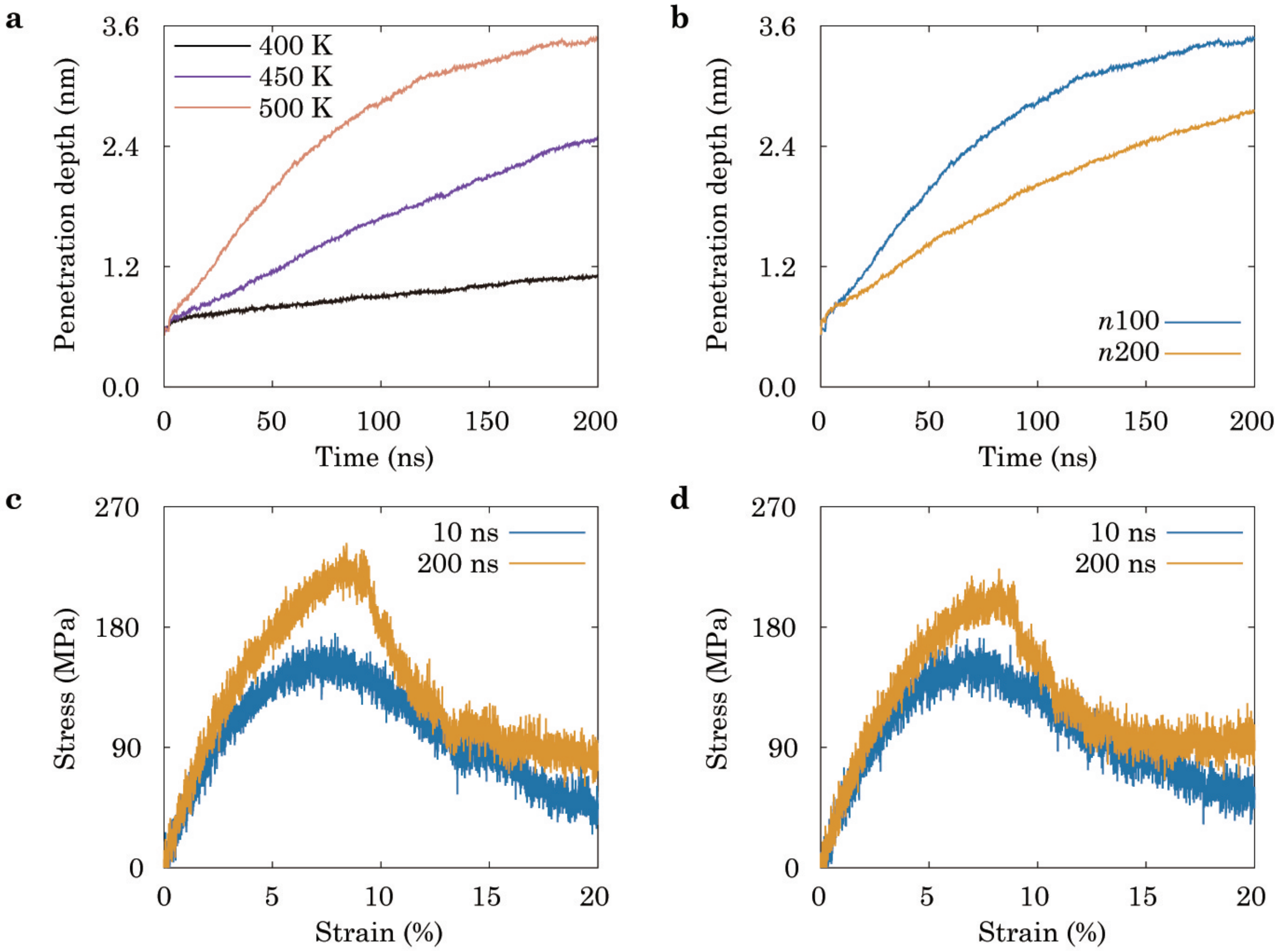

Figure S2. CGMD simulation results of the penetration depth of the PE/PP interfaces at different (**a**) fusion temperature ($T = 400, 450, 500$ K) and (**b**) chain lengths (n100 and n200). Stress-strain curves for the CG models with chain lengths (**c**) n100 and (**d**) n200, where the time of fusion is 10 and 200 ns.

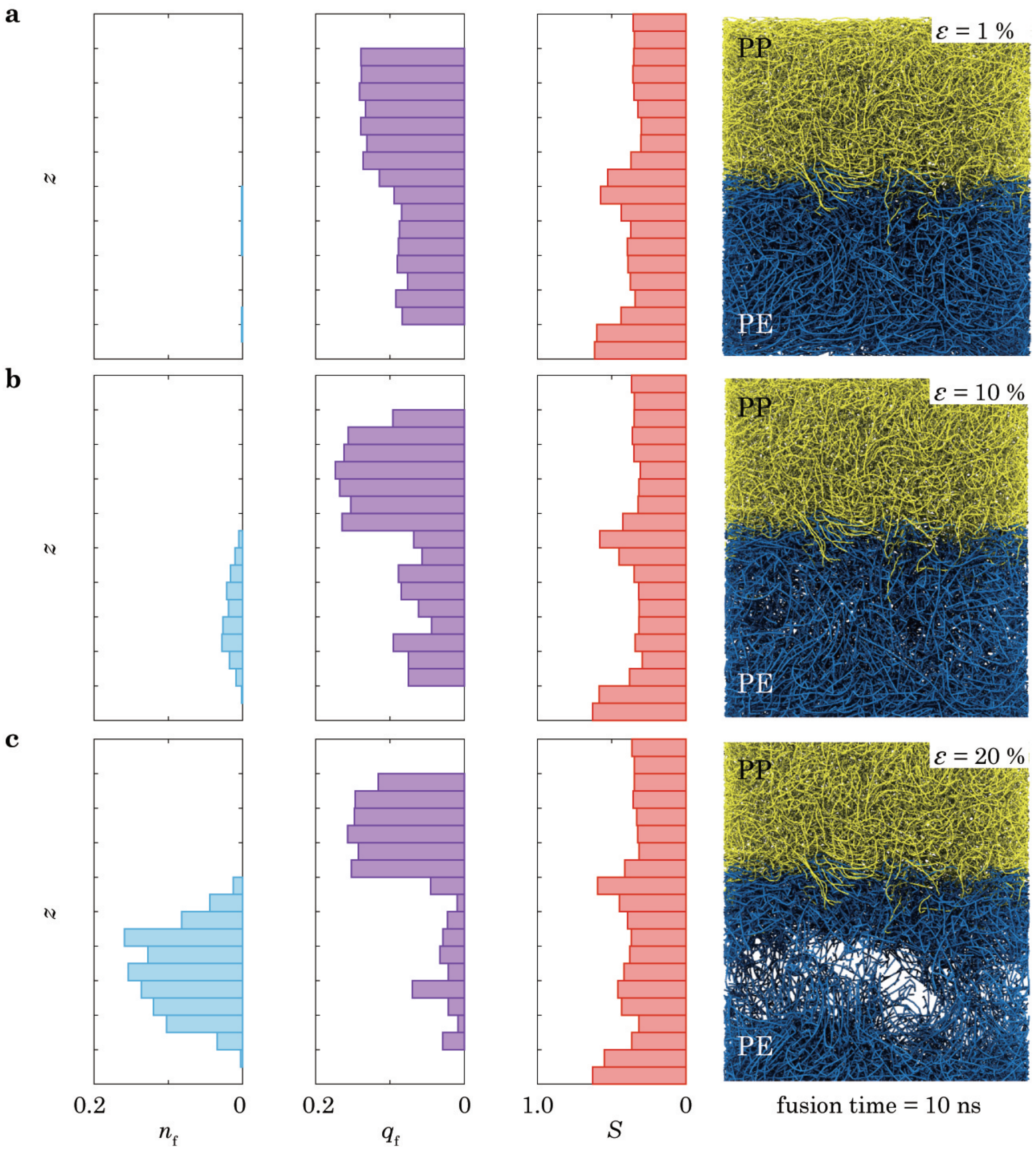

Figure S3. Microstructural evolution of the PE/PP interfaces under tensile strain of (**a**) $\varepsilon = 1\%$, (**b**) 10%, (**c**) 20% in the CGMD simulations. The three columns are the spatial distributions, in the direction normal to the interface, of the fraction of fractured bonds n_f, the load filling factor q_f, and the order parameter S from the left to the right. The PE/PP interface is fused for 10 ns, at 500 K and 1 atm.

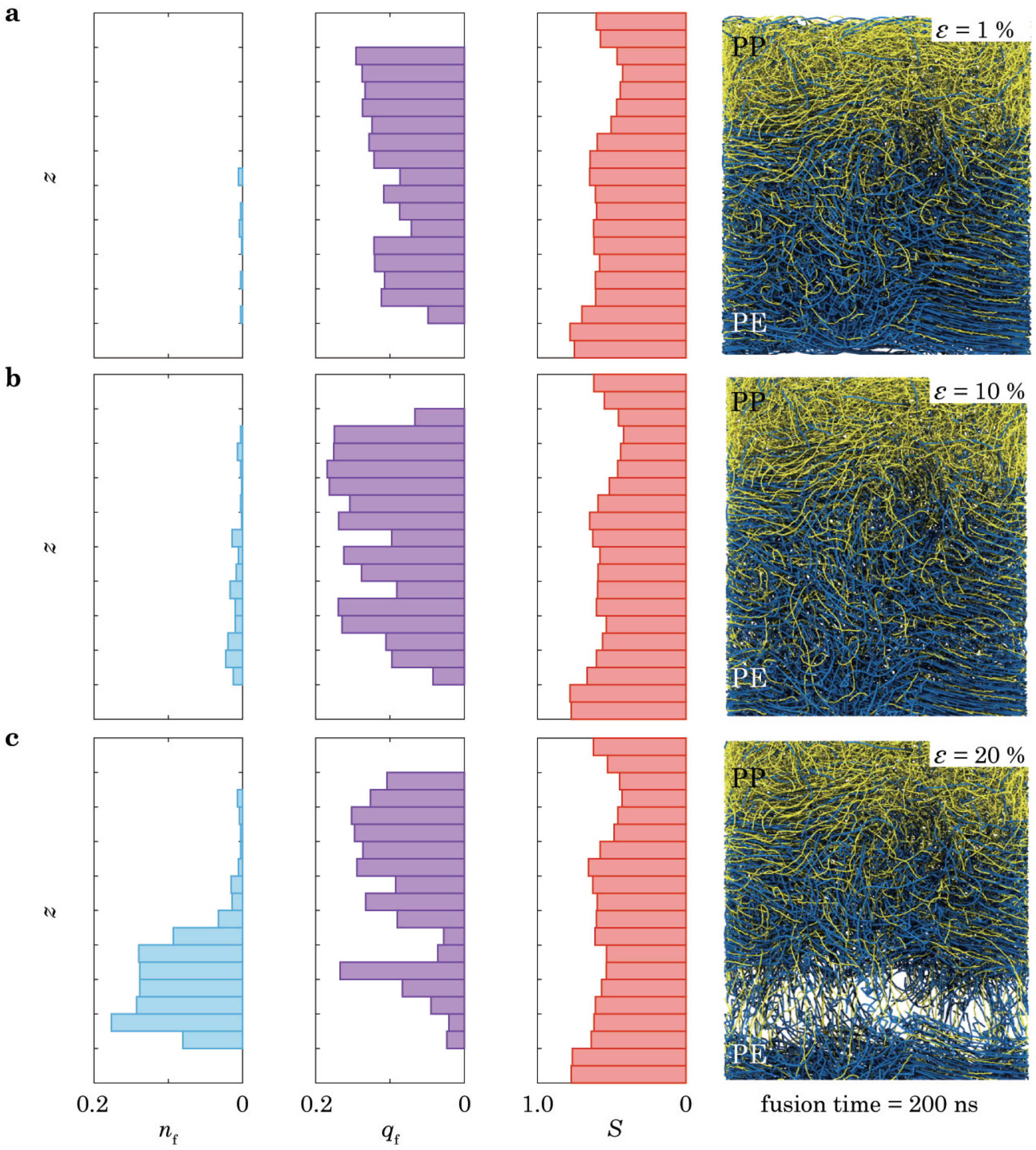

Figure S4. Microstructural evolution of the PE/PP interfaces under tensile strain of (**a**) $\varepsilon = 1\%$, (**b**) 10%, (**c**) 20% in the CGMD simulations. The three columns are the spatial distributions, in the direction normal to the interface, of the fraction of fractured bonds n_f, the load filling factor q_f, and the order parameter S from the left to the right. The PE/PP interface is fused for 200 ns, at 500 K and 1 atm.

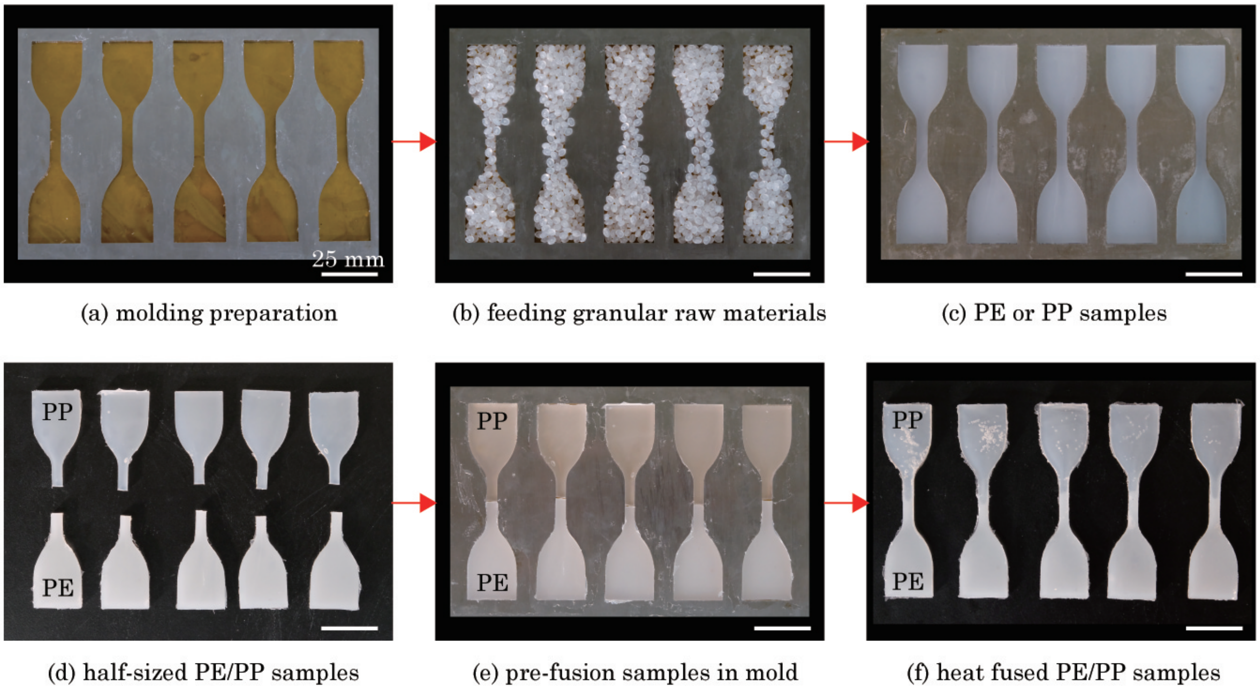

Figure S5. Flow charts of hot-compression molding (HCM) employed to manufacture fused PE/PP samples.

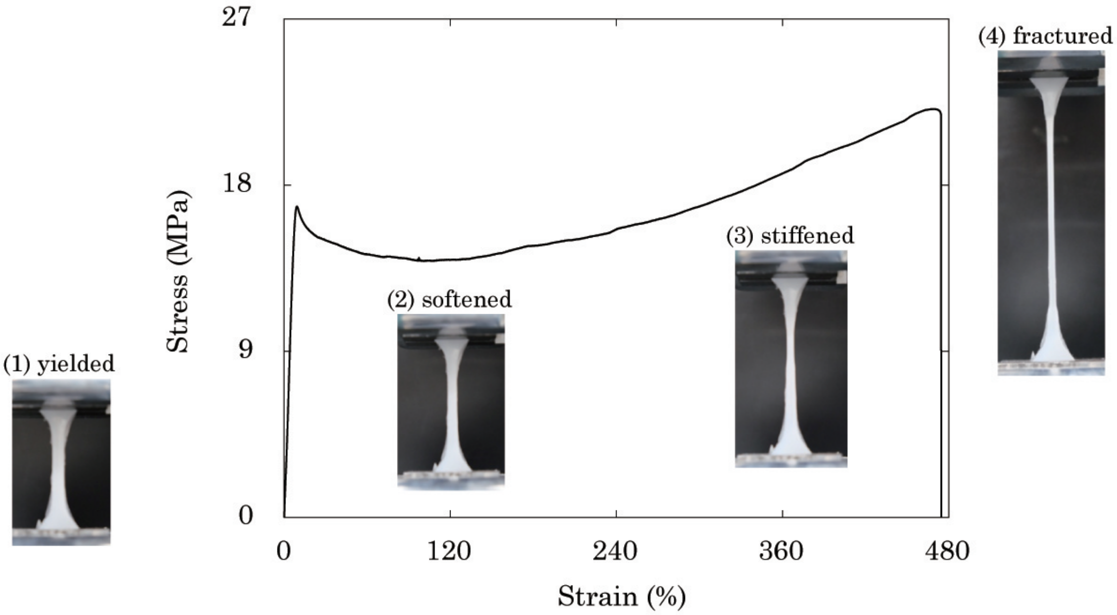

Figure S6. A typical stress-strain curve of the pure PP-2 sample in the experiments.

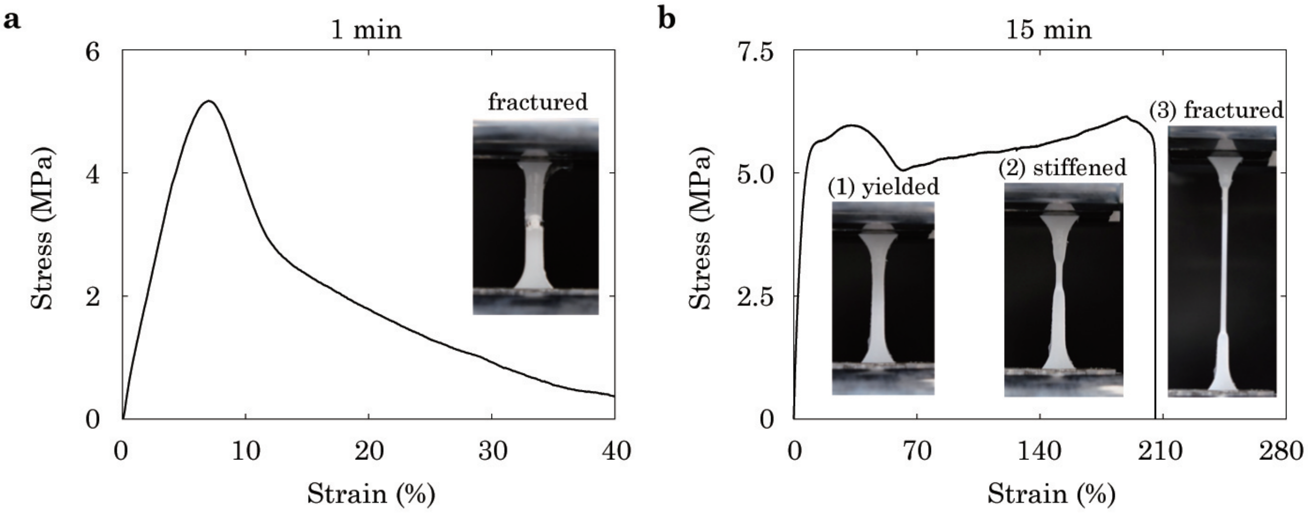

Figure S7. Stress-strain curves for the fused LLDPE/PP-1 samples obtained with (**a**) shorter (1 min) and (**b**) longer fusion time (15 min) in the experiments. The fusion temperature is 180 °C.

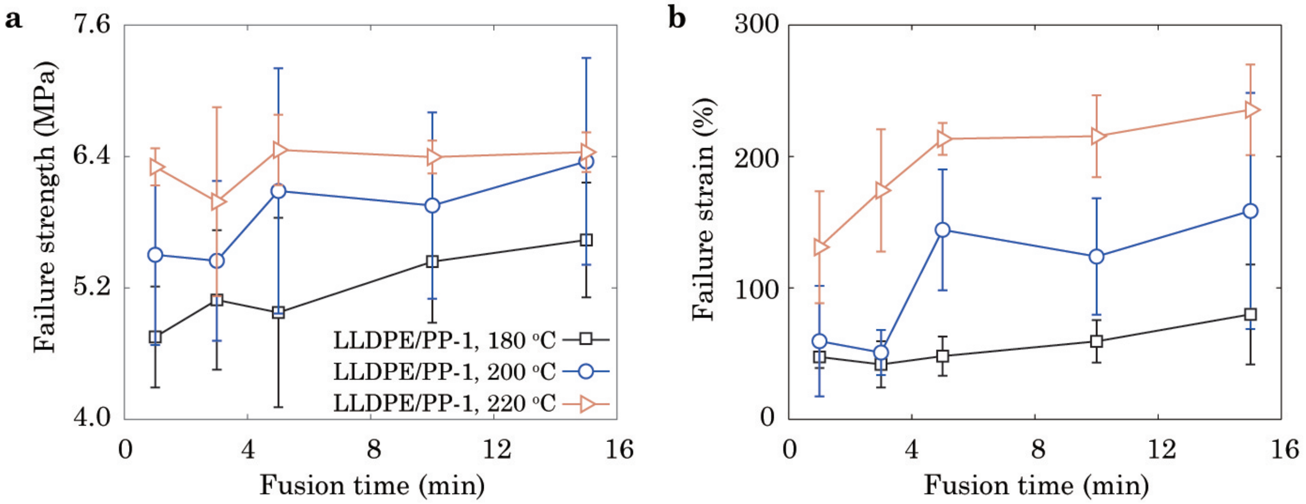

Figure S8. The experimental results of (**a**) the failure strength and (**b**) strain to failure of the LLDPE/PP-1 samples measured at different fusion temperature ($T = 180,\ 200,\ 220\ ^{\rm o}$C).

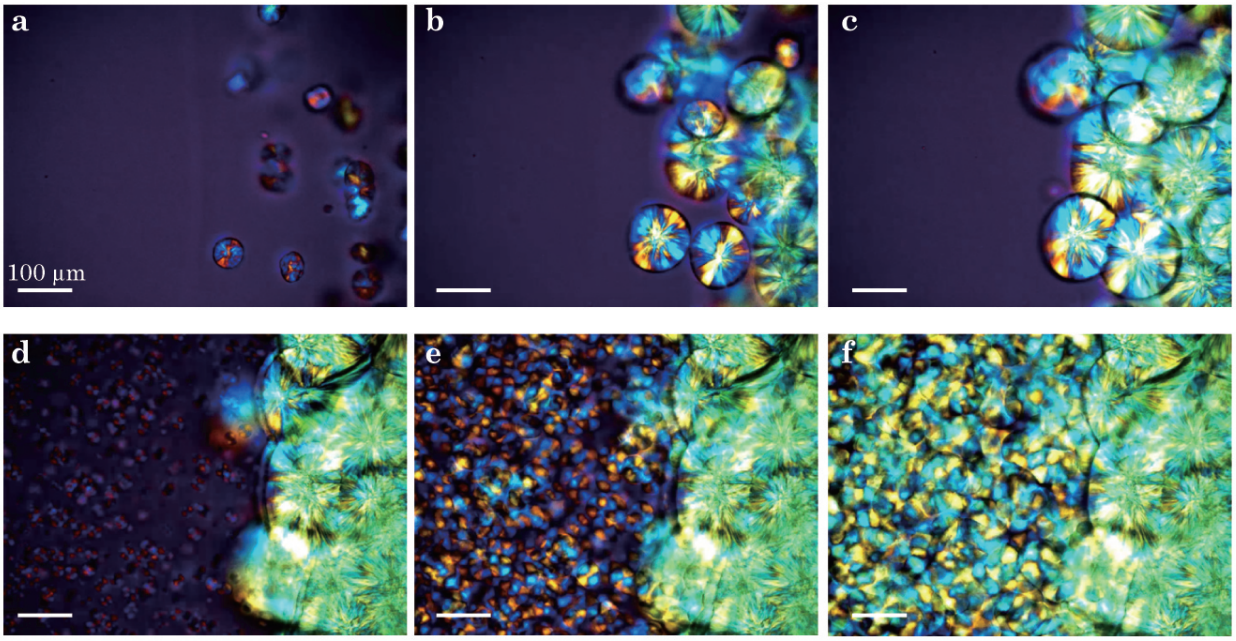

Figure S9. Isothermal crystallization at the LLDPE/PP-1 interfaces in the experiments. Crystallizations of (**a**-**c**) PP-1 (right) at $\sim$ 130 °C and (**d**-**f**) LLDPE (left) at $\sim$ 110 °C. The crystallizations for LLDPE and PP-1 start separately, and the interface does not induce the nucleation process of LLDPE or PP-1, suggesting that the crystallization has a minor effect on the degree of fusion. The scale bar is 100 μm.

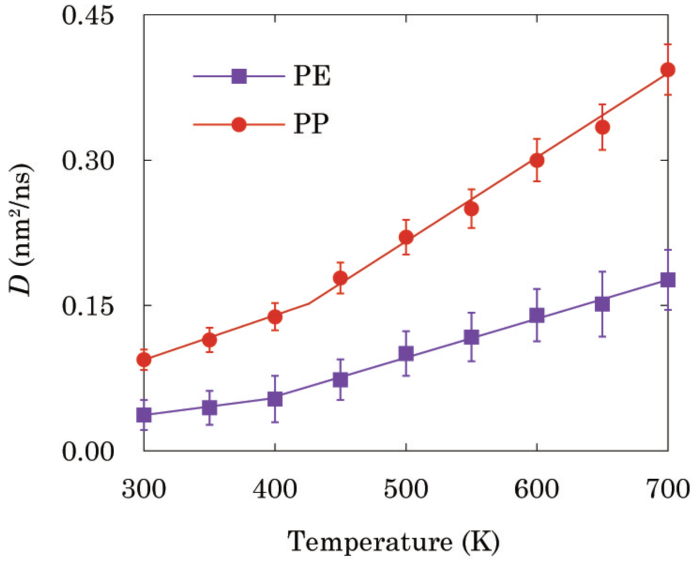

Figure S10. Coefficients of diffusion, D, plotted as functions of temperature for PE and PP as predicted by the coarse-grained molecular dynamics (CGMD) simulations. The length (n100) indicates 100 beads of PE and 130 beads of PP in the CG model. The pressure is 1 atm.
